# Supplementary material for: Structures of a sperm-specific solute carrier gated by voltage and cAMP
Source: Nature. 2023 Oct 25;623(7985):202–9. doi: 10.1038/s41586-023-06629-w (PMC10620091; doi:10.1038/s41586-023-06629-w)
Supplement: Supplementary file 1 — This file contains Supplementary Figures 1 & 2 and legends for Supplementary Video files. [file 41586_2023_6629_MOESM1_ESM.pdf]

nature portfolio

https://doi.org/10.1038/s41586-023-06629-w

Supplementary information

Structures of a sperm-specific solute carrier gated by voltage and cAMP

In the format provided by the authors and unedited

Supplementary Information Figure 1. Uncropped SDS-PAGE gel of purified SpSLC9C1 assembled into lipid nanodiscs displayed in Extended Data Figure 1b. Dashed line indicates the part of the image displayed in Extended Data Figure 1b, molecular weight of the marker (PageRuler Plus, Thermo Fisher Scientific) is indicated.

Supplementary Information Figure 2. Sequence alignment of SLC9C1 select homologs. Represented are: echinoderms (SpSLC9C1, NP_001091927.1), fishes (spotted gar, XP_015193550.1; salmon, XP_013979929.1), reptiles (green sea turtle, XP_027674929.1; tiger snake, XP_026524154.1) and mammals (mouse, NP_932774.3; human, NP_898884.1). Secondary structure elements are indicated, individual domains are displayed in unique colors – dimer and core domains of the transport domain (TD) are in green and light blue, respectively, voltage sensing domain (VSD) in dark blue, coupling helices (CH) in yellow, CNBD in light-purple, C-terminal β-roll (β-CTD) in dark purple. Residues involved in ion coordination are shown in orange, gating charges on S4 in blue, 'lid' on the gating charge transfer center (GCTC) in yellow, countercharges of the GCTC in red.

Supplementary Video 1. Conformational heterogeneity of the cytoplasmic domain identified in the 3D variability analysis of the dimeric cAMP-bound SpSLC9C1.

Supplementary Video 2. Conformational heterogeneity of cAMP-bound SpSLC9C1 on a monomer level. Morph between two protomer maps identified in the cAMP-bound dataset. Protomer state 1is shown in orange, protomer state 2 in yellow, selected helices are indicated.

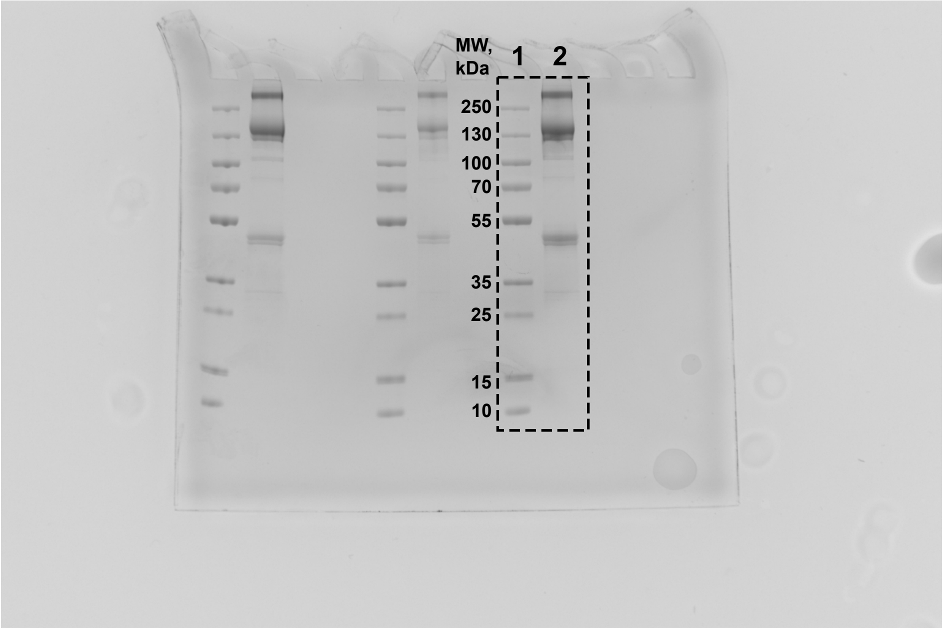

1 - marker
2 - final concentrated SpSLC9C1 sample

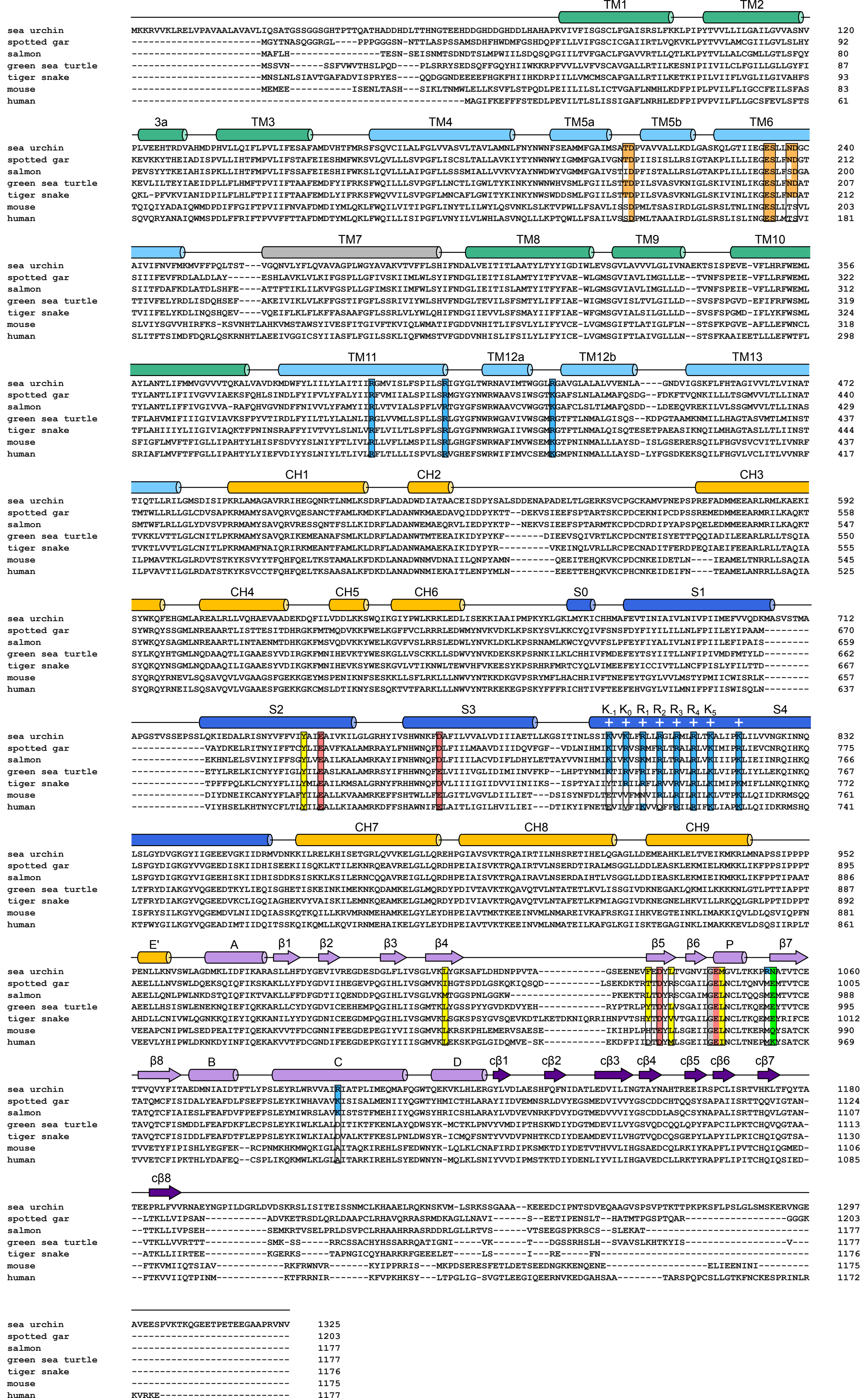

TM1
TM2
sea urchin 120
spotted gar 92
salmon 80
green sea turtle 87
tiger snake 93
mouse 83
human 61
3a
TM3
TM4
TM5a
TM5b
TM6
sea urchin 240
spotted gar 212
salmon 200
green sea turtle 207
tiger snake 212
mouse 203
human 181
TM7
TM8
TM9
TM10
sea urchin 356
spotted gar 322
salmon 312
green sea turtle 319
tiger snake 324
mouse 318
human 298
TM11
TM12a
TM12b
TM13
sea urchin 472
spotted gar 440
salmon 429
green sea turtle 437
tiger snake 444
mouse 437
human 417
CH1
CH2
CH3
sea urchin 592
spotted gar 558
salmon 547
green sea turtle 550
tiger snake 555
mouse 545
human 525
CH4
CH5
CH6
S0
S1
sea urchin 712
spotted gar 670
salmon 659
green sea turtle 662
tiger snake 667
mouse 657
human 637
S2
S3
K-1 K0 R1 R2 R3 R4 K5
S4
sea urchin 832
spotted gar 775
salmon 766
green sea turtle 767
tiger snake 772
mouse 761
human 741
CH7
CH8
CH9
sea urchin 952
spotted gar 895
salmon 886
green sea turtle 887
tiger snake 892
mouse 881
human 861
E'
A
β1
β2
β3
β4
β5
β6
P
β7
sea urchin 1060
spotted gar 1005
salmon 988
green sea turtle 995
tiger snake 1012
mouse 990
human 969
β8
B
C
D
cβ1
cβ2
cβ3
cβ4
cβ5
cβ6
cβ7
sea urchin 1180
spotted gar 1124
salmon 1107
green sea turtle 1113
tiger snake 1130
mouse 1106
human 1085
cβ8
sea urchin 1297
spotted gar 1203
salmon 1177
green sea turtle 1177
tiger snake 1176
mouse 1175
human 1172
sea urchin AVEESPVKTKQGEETPETEEGAAPRVNV 1325
spotted gar 1203
salmon 1177
green sea turtle 1177
tiger snake 1176
mouse 1175
human KVRKE 1177
