# Supplementary material for: Structures of a sperm-specific solute carrier gated by voltage and cAMP
Source: Nature. 2023 Oct 25;623(7985):202–9. doi: 10.1038/s41586-023-06629-w (PMC10620091; doi:10.1038/s41586-023-06629-w)
Supplement: Supplementary file 2 — Reporting Summary [file 41586_2023_6629_MOESM2_ESM.pdf]

nature portfolio

Corresponding author(s): C. Paulino

Last updated by author(s): Aug 23, 2023

Reporting Summary

Nature Portfolio wishes to improve the reproducibility of the work that we publish. This form provides structure for consistency and transparency in reporting. For further information on Nature Portfolio policies, see our Editorial Policies and the Editorial Policy Checklist.

Statistics

For all statistical analyses, confirm that the following items are present in the figure legend, table legend, main text, or Methods section.

n/a	Confirmed	
☐	☒	The exact sample size (*n*) for each experimental group/condition, given as a discrete number and unit of measurement
☐	☒	A statement on whether measurements were taken from distinct samples or whether the same sample was measured repeatedly
☒	☐	The statistical test(s) used AND whether they are one- or two-sided<br>*Only common tests should be described solely by name; describe more complex techniques in the Methods section.*
☒	☐	A description of all covariates tested
☒	☐	A description of any assumptions or corrections, such as tests of normality and adjustment for multiple comparisons
☐	☒	A full description of the statistical parameters including central tendency (e.g. means) or other basic estimates (e.g. regression coefficient) AND variation (e.g. standard deviation) or associated estimates of uncertainty (e.g. confidence intervals)
☒	☐	For null hypothesis testing, the test statistic (e.g. *F*, *t*, *r*) with confidence intervals, effect sizes, degrees of freedom and *P* value noted<br>*Give P values as exact values whenever suitable.*
☒	☐	For Bayesian analysis, information on the choice of priors and Markov chain Monte Carlo settings
☒	☐	For hierarchical and complex designs, identification of the appropriate level for tests and full reporting of outcomes
☒	☐	Estimates of effect sizes (e.g. Cohen's *d*, Pearson's *r*), indicating how they were calculated

Our web collection on statistics for biologists contains articles on many of the points above.

Software and code

Policy information about availability of computer code

Data collection	Cryo-EM: SerialEM 3.8.0 beta or 3.9.0 beta, EPU 2.7.0 or 2.8.0 (Thermo Fisher Scientific). ITC: MicroCal PEAQ-ITC Control and Analysis v1.41. NanoDSF: Prometheus Panta.Control 1.4.3 and Panta.Analysis 1.4.3
Data analysis	Cryo-EM (managed through SBGrid version 2.5.6): cryoSPARC v3, Relion 3.1.0, Focus 1.1.0, MotionCor2 version 1.4.0, CTFfind4.1.14, crYOLO 1.7.5, 1.7.6 or 1.8.2, coot version 0.9.8.1, Phenix 1.20.1-4487, Isolde 1.6.0, deepEMhancer 20220530_cu10, Alphafold2 v2.3.2, Pymol 2.5.5, ChimeraX 1.6.1, Chimera 1.17.3

For manuscripts utilizing custom algorithms or software that are central to the research but not yet described in published literature, software must be made available to editors and reviewers. We strongly encourage code deposition in a community repository (e.g. GitHub). See the Nature Portfolio guidelines for submitting code & software for further information.

Data

Policy information about availability of data

All manuscripts must include a data availability statement. This statement should provide the following information, where applicable:

- Accession codes, unique identifiers, or web links for publicly available datasets
- A description of any restrictions on data availability
- For clinical datasets or third party data, please ensure that the statement adheres to our policy

Data supporting the findings of this manuscript are available from the corresponding authors upon request. Cryo-EM density maps, half maps, and masks have been

deposited in the Electron Microscopy Data Bank (EMDB), atomic models are available through the Protein Data Bank (PDB), the data are available under the following accession codes: detergent-solubilized apo SpSLC9C1 symmetric class (EMDB:17603) and asymmetric class (EMDB:17604), nanodisc reconstituted apo SpSLC9C1 dimeric (PDB:8PCZ, EMDB:17596), apo SpSLC9C1 protomer state 1 (PDB:8PD2, EMDB:17598), apo SpSLC9C1 protomer state 2 (PDB:8PD3, EMDB:17599), apo SpSLC9C1 protomer state 3 (PDB:8PD5, EMDB:17601), apo SpSLC9C1 protomer state 4 (PDB:8PD7, EMDB:17602), cGMP-bound dimeric (PDB:8PDU, EMDB:17621), cGMP-bound protomer (PDB:8PDV, EMDB:17622), cAMP-bound dimeric (PDB:8PD8, EMDB:17605), cAMP-bound protomer state 1 (PDB:8PD9, EMDB:17607), cAMP-bound protomer state 2 (EMDB:17625). Micrographs were deposited to EMPIAR under the following accession codes: EMPIAR-11628 (ligand-free SpSLC9C1 in nanodiscs), EMPIAR-11629 (ligand-free SpSLC9C1 in detergent), EMPIAR-11635 (cAMP-bound SpSLC9C1 in lipid nanodiscs), and EMPIAR-11630 (cGMP-bound SpSLC9C1 in lipid nanodiscs). Following amino acid sequences were used for sequence alignments and are available from Uniprot: SpSLC9C1, NP_001091927.1; spotted gar, XP_015193550.1; salmon, XP_013979929.1,; green sea turtle, XP_027674929.1; tiger snake, XP_026524154.1; mouse, NP_932774.3; human, NP_898884.1. Following protein structures were used in this study and are available in the PDB: 5U6O, 5JON, 3U10, 3BPZ, 4CZB, 6Z3Z, 7P1K, 6V1X, 7SIP, 8PD2, 7NP3, 6CJQ, 5U6P, 7NP4, 6Z3Y and 3J4Q.

Research involving human participants, their data, or biological material

Policy information about studies with human participants or human data. See also policy information about sex, gender (identity/presentation), and sexual orientation and race, ethnicity and racism.

Reporting on sex and gender	N/A
Reporting on race, ethnicity, or other socially relevant groupings	N/A
Population characteristics	N/A
Recruitment	N/A
Ethics oversight	N/A

Note that full information on the approval of the study protocol must also be provided in the manuscript.

Field-specific reporting

Please select the one below that is the best fit for your research. If you are not sure, read the appropriate sections before making your selection.

☒ Life sciences ☐ Behavioural & social sciences ☐ Ecological, evolutionary & environmental sciences

For a reference copy of the document with all sections, see nature.com/documents/nr-reporting-summary-flat.pdf

Life sciences study design

All studies must disclose on these points even when the disclosure is negative.

Sample size	No statistical methods were used to predetermine the sample size. Cryo-EM data collection was deemed sufficient if it was possible to classify the structural heterogeneity and to reach the resolution better than 4 Å in all of the subsequent 3D reconstructions originating from the same dataset. For ITC and nanoDSF experiments, the sample size was chosen according to the standard in the field.
Data exclusions	According to the standard in the cryo-EM field, micrographs with the resolution worse than 6 Å, displaying ice contamination/cracks, unusually high drift values, defocus values > 2 um for data collected at 200 kV, or > 3 um for the data collected at 300 kV, were excluded from the subsequent data analysis. Similarly, particles were excluded during 2D classification if they did not produce well-resolved classes with identifiable protein density, and during 3D classification, if they did not contribute to the 3D reconstructions with all of the protein domains resolved, and did not yield high-resolution 3D classes. For ITC and nanoDSF measurements, no data exclusion criteria were established prior to the data collection.
Replication	For ITC experiments, three biological replicates were measured. For nanoDSF experiments, three technical replicates were measured. All attempts at replication were successful. Protein was purified and reconstituted into nanodiscs >3 times with similar results. Structure determination was performed once per given dataset, for ligand-bound datasets several grids were imaged, producing micrographs of similar quality and appearance.
Randomization	Particles were randomized between even/odd groups during refinement and resolution estimation (gold-standard FSC).
Blinding	Blinding criteria are not applicable to cryo-EM data processing, as the data is handled in an automated fashion.

Reporting for specific materials, systems and methods

We require information from authors about some types of materials, experimental systems and methods used in many studies. Here, indicate whether each material, system or method listed is relevant to your study. If you are not sure if a list item applies to your research, read the appropriate section before selecting a response.

Materials & experimental systems

n/a	Involved in the study
☒	☐ Antibodies
☐	☒ Eukaryotic cell lines
☒	☐ Palaeontology and archaeology
☒	☐ Animals and other organisms
☒	☐ Clinical data
☒	☐ Dual use research of concern
☒	☐ Plants

Methods

n/a	Involved in the study
☒	☐ ChIP-seq
☒	☐ Flow cytometry
☒	☐ MRI-based neuroimaging

Eukaryotic cell lines

Policy information about cell lines and Sex and Gender in Research

Cell line source(s)	All of the cell lines used are commercially available: HEK293S GnTI- (CRL-3022, ATCC), Sf9 (12659017,ThermoFisher Scientific)
Authentication	No further authentication was performed after purchasing the cell lines
Mycoplasma contamination	All cell lines were tested for Mycoplasma every 3-4 months and were found negative
Commonly misidentified lines (See ICLAC register)	N/A
